# Multimodal magnetic resonance spectroscopy and surface-based morphometry study of individuals at ultra-high-risk for psychosis

**DOI:** 10.1192/j.eurpsy.2021.362

**Published:** 2021-08-13

**Authors:** A. Tomyshev, P. Menshchikov, M. Omelchenko, V. Kaleda, D. Kupriyanov, I. Lebedeva

**Affiliations:** 1 Laboratory Of neuroimaging And Multimodal Analysis, FSBSI Mental Health Research Center, Moscow, Russian Federation; 2 Clinical Science, LLC Philips Healthcare Russia, Moscow, Russian Federation; 3 Department Of Youth Psychiatry, FSBSI Mental Health Research Center, Moscow, Russian Federation; 4 Department Of Endogenous Mental Disorders, FSBSI Mental Health Research Center, Moscow, Russian Federation; 5 Neurocognitive Research Department, National Research Center “Kurchatov Institute”, Moscow, Russian Federation

**Keywords:** Magnetic resonance spectroscopy, GABA, Cortical thickness, Ultra-high risk of psychosis

## Abstract

**Introduction:**

Studies examining gamma-aminobutyric acid (GABA) or glutamate in ultra-high risk for psychosis (UHR) have shown conflicting results, and a number of multimodal studies examining associations between metabolite and structural characteristics is very limited.

**Objectives:**

We aimed to investigate potential associations between GABA and glutamate levels and cortical thickness in the frontal lobe in UHR individuals and healthy controls (HC).

**Methods:**

20 male UHR individuals and 19 healthy controls (HC) underwent structural MRI and MR spectroscopy at 3T Philips scanner. T1-weighted images were processed via FreeSurfer 6.0 to quantify cortical thickness for selected frontal regions labeled according to Desikan atlas. MEGA-PRESS acquisitions were analyzed with jMRUi (ver. 5.1 Alpha), levels of GABA and glutamate were calculated as ratios to creatine + phosphocreatine.

**Results:**

The study revealed: 1) GABA/Cr ratios reduction in the left frontal lobe (p=0.001) which was not attributable to antipsychotic medication; 2) cortical thickness reductions in the left pars orbitalis (p=0.005) (the anterior part of the inferior frontal gyrus) in the UHR individuals compared to HC. No significant correlations between GABA/Cr ratios and cortical thickness were identified in both groups.

**Conclusions:**

The findings indicate that the UHR state is associated with altered GABA levels and cortical thickness reductions in the prefrontal cortex. The results also show that GABA levels are not directly related to cortical abnormalities, suggesting that altered metabolite levels may be associated with a complex system of structural and functional impairments, rather than directly correlating with structural changes in separate cortical regions. The work was supported by RFBR grant 19-29-10040.

**Disclosure:**

No significant relationships.

